# A new species related to *Anorthoachangi* from Wuyishan National Park (Lepidoptera, Noctuidae, Hadeninae)

**DOI:** 10.3897/BDJ.12.e139425

**Published:** 2024-12-06

**Authors:** Rui Xie, Liang Guo, Chenbin Wang, Hongdi Gao

**Affiliations:** 1 Nanjing Institute of Environmental Sciences, Ministry of Ecology and Environment; National Key Laboratory of Biosafety, Nanjing, China Nanjing Institute of Environmental Sciences, Ministry of Ecology and Environment; National Key Laboratory of Biosafety Nanjing China; 2 State Environmental Protection Scientific Observation and Research Station for Ecology and Environment of Wuyi Mountains, Ministry of Ecology and Environment, Nanjing, China State Environmental Protection Scientific Observation and Research Station for Ecology and Environment of Wuyi Mountains, Ministry of Ecology and Environment Nanjing China; 3 Department of Entomology, College of Plant Protection, South China Agricultural University, Guangzhou, China Department of Entomology, College of Plant Protection, South China Agricultural University Guangzhou China; 4 Zhejiang Forest Resources Monitoring Center, Hangzhou, China Zhejiang Forest Resources Monitoring Center Hangzhou China

**Keywords:** *Anorthoa*, Orthosiini, *rubrocinerea*-group, taxonomy, China

## Abstract

**Background:**

*Anorthoa* Berio, 1980 is a genus within the subfamily Hadeninae . This genus has a close relationship with the *rama* species complex of the genus *Harutaeographa*. It comprises three species groups: the *munda*-group, the *angustipennis*-group and the *rubrocinerea*-group. The species *Anorthoachangi* Ronkay & Ronkay, 2001 from Taiwan Island belongs to the *rubrocinerea*-group.

**New information:**

A new species related to *Anorthoachangi* Ronkay & Ronkay, 2001, is described from Wuyishan National Park, China. The new species can be distinguished from its sister species by its larger size and some differences in the genitalia. Now, the number of species in the genus Anorthoa is increased to eleven.

## Introduction

The Hadenid genus *Anorthoa* was originally established as a subgenus of the genus *Orthosia* Ochsenheimer, 1816 with *Noctuamunda* [Denis & Schiffermüller], 1775 as the type species (Berio 1980). Subsequently, *Anorthoa* was recognised as an independent genus and subdivided into three species groups: the *munda*-group, the *angustipennis*-group and the *rubrocinerea*-group. Currently, ten species are known in the genus (Ronkay et al. 2001, Ronkay et al. 2010, Owada et al. 2015, Benedek and Tóth 2021). The *rubrocinerea*-group consists of three species: *A.rubrocinerea* (Hreblay & Ronkay, 1998), *A.changi* Ronkay & Ronkay, 2001 and *A.biborka* Ronkay, Ronkay, Gyulai & Hacker, 2010 (Hreblay and Ronkay 1998, Hreblay and Ronkay 1999, Ronkay and Ronkay 2001, Ronkay et al. 2010). In this paper, a new species related to *A.changi* from Wuyishan National Park is described and illustrated.

## Materials and methods

Living moths were collected using a 450W high-pressure mercury vapour lamp in Wuyishan National Park, Wuyishan City, Fujian Province, China and were treated with ammonia immediately. Photographs of the living adults were taken using a Canon EOS 200D digital camera with a SIGMA 105 mm 1:2.8 DG MACRO HSM lens, and the specimens were photographed using a Nikon D7100 digital camera with a LAOWA 60mm f/2.8 2X Ultra-Macro lens. Then, the abdomens were removed and macerated in hot 10% sodium hydroxide (NaOH) solution for the examination of genitalia. Photos of genitalia were taken under a Carl Zeiss Discovery V12 digital microscope. All photos were processed using Adobe Photoshop CS5® software. The specimens examined in this study are deposited in South China Agricultural University (SCAU), Guangzhou City, Guangdong Province, China.

## Taxon treatments

### Anorthoa wangi

Guo & Xie sp. nov.

B9BC0130-B3E2-57C6-915F-04FF90F15CE4

#### Materials

- **Type status:** Holotype. **Occurrence:** recordedBy: Tengda Liang, Yulong Zhang & Feiran Chen; sex: male; occurrenceID: D907DE2A-845D-5BCE-A230-6CF15720C4CB; **Location:** country: China; stateProvince: Fujian; locality: Nanping City, Wuyishan City, Wuyishan National Park, the observation tower of Xianfengling; verbatimElevation: 1200 m; verbatimLatitude: 27°42′39.29″N; verbatimLongitude: 117°39′8.64″E; **Event:** eventDate: 04-03-2023
- **Type status:** Paratype. **Occurrence:** recordedBy: Tengda Liang, Yulong Zhang & Feiran Chen; sex: 5males, 1female; occurrenceID: 032E7781-19BC-56A0-87FB-055F89F9FBE2; **Location:** country: China; stateProvince: Fujian; locality: Nanping City, Wuyishan City, Wuyishan National Park, the observation tower of Xianfengling; verbatimElevation: 1200 m; verbatimLatitude: 27°42′39.29″N; verbatimLongitude: 117°39′8.64″E; **Event:** eventDate: 05-03-2023

#### Description

**Figure 2.**
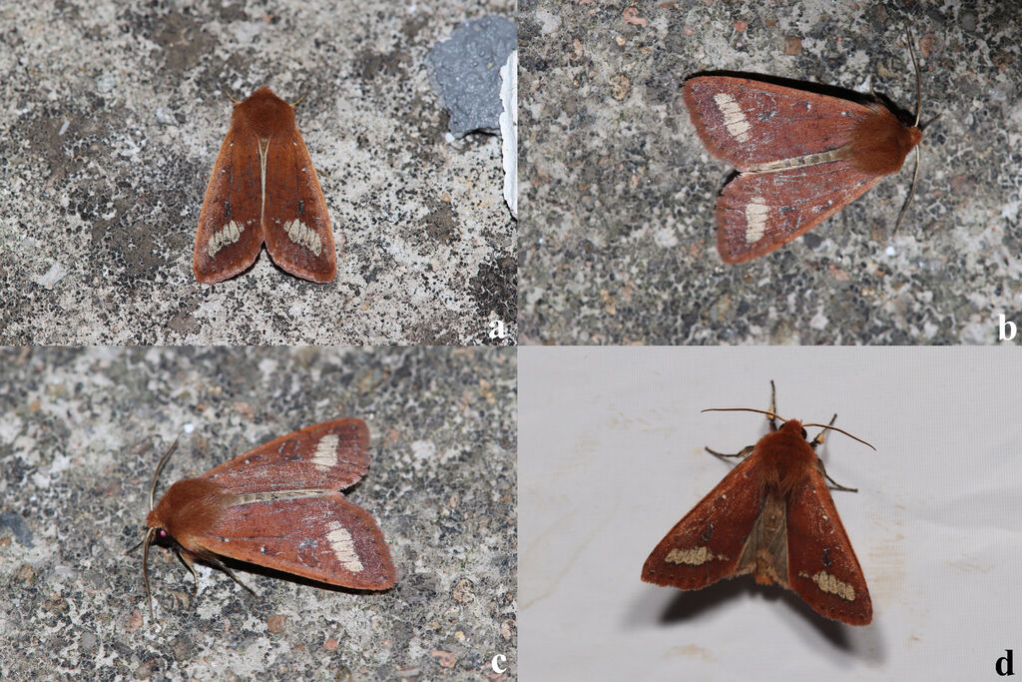
Live male adults of *Anorthoawangi* **sp. nov.** from Fujian, China (a-c represent the same individual). Photo by Yulong Zhang.

**Male** (Fig. 1a, b, c and Fig. 2). Forewing length 17-18 mm (n = 6, 17 mm in holotype), wingspan 36-38 mm (n = 6, 36 mm in holotype). Head small, densely covered with pale rufous brown hairs, labial palpus well-developed, outer side dark brown, antenna finely dentate, fascicularly ciliate, deep ochreous brown mainly, paler basally, compound eyes hemispherical, surface ciliate. Thorax dorsally deep ochreous brown or rufous brown, collar and tegula the same colour. Fore-wing nearly triangular, narrow and elongate, apex pointed; forewing ground colour deep ochreous brown or rubiginous, costa paler, except apical angle. A dark brown sub-basal dot present with whitish scales, antemedial line, postmedial line and median fascia obscure, nearly disappearing or present as pale brownish-greyish shadows. Orbicular and reniform stigmata nearly unrecognisable in some specimens, outlines of the recognisable stigmata brownish with whitish scales, filling darker brown than ground colour. The area between postmedial and subterminal lines widely pale ochreous, suffused with dark brown scales in the area near the subterminal line. Subterminal line brown, more or less sinuous, terminal line present as a row of brown spots with a few whitish scales, cilia as ground colour. Hindwing pale ochreous, with weak pale brownish suffusion, discal spot present, cilia as ground colour.

**Female** (Fig. 1d). Forewing length 18 mm, wingspan 39 mm. Similar to male, but antenna filiform, shortly ciliate, forewing somewhat more rounded.

**Male genitalia** (Fig. 3). Uncus short and lanceolate. Tegumen normal, short and narrow, with weak penicular lobes. Fultura inferior sclerotised, nearly in the shape of an arc-sided inverted triangle. Transtilla heavily sclerotised, falcate and equipped with strong teeth. Saccus well-developed, V-shaped, narrow and elongate. Valva elongated, but significantly shorter than the aedeagus, evenly tapering towards a small, triangular, apically finely pointed cucullus. Corona undeveloped, pollex short and small. Sacculus broad and clavus reduced. Ampulla well-developed, arc-shaped, basally thick and apically digitiform. Harpe flattened, trapezoidal-capitiform with a long basal bar. Aedeagus cylindrical, long and thick, ventral carina bar eversible, quite long and sclerotised, ending with several acute teeth, ventro-lateral carina bar short, ending with a triangular tooth. Vesica quite long, with a tortuous tubular shape, the sub-basal diverticulum of the basal part of the vesica bears a cluster of spiculiform cornuti, distal part of the vesica armed with a long field of small, spiculiform cornuti.

**Figure 4.**
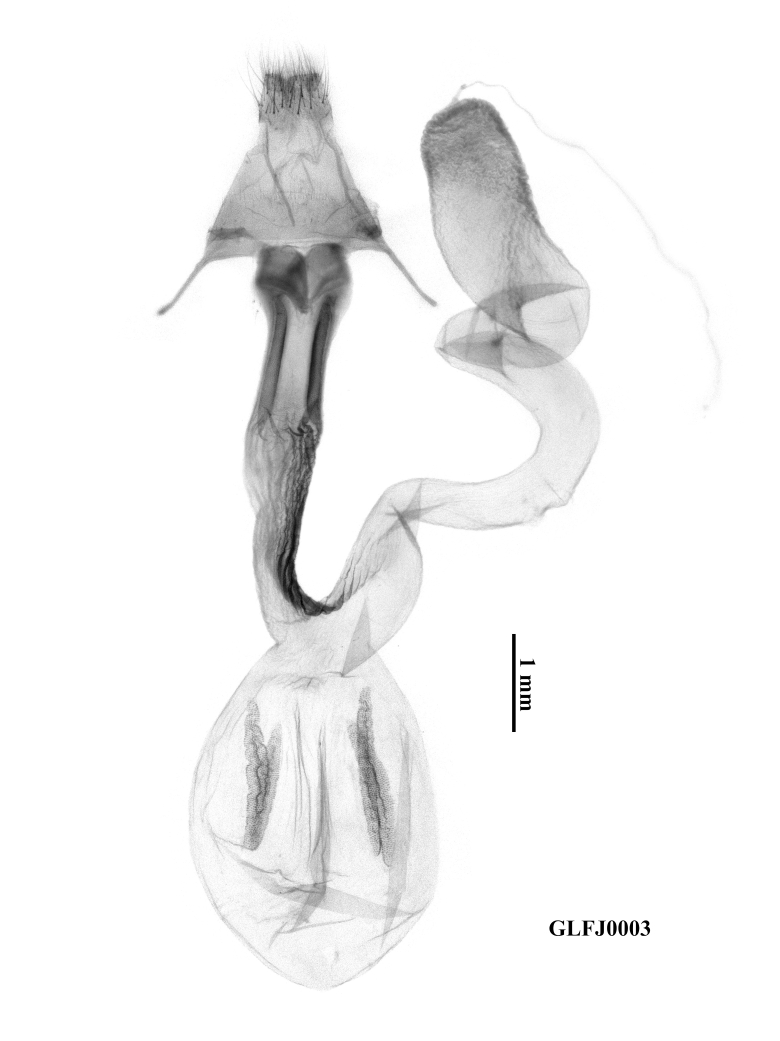
Female genitalia of *Anorthoawangi* **sp. nov.**, paratype, Fujian, China (prep. GLFJ0003). Scale bar = 1 mm.

**Female genitalia** (Fig. 4). Papillae anales trapezoidal. Apophyses posteriores and anteriores normal, both slender, with the former slightly longer than the latter. Ostium bursae tubular, long and narrow and the anterior three-quarters sclerotised, with heavily sclerotised narrow marginal folds on the dorsal side. The proximal end of the ostium fused with the ductus by a ribbed-cristate ring. Ductus bursae narrowly tubular, densely wrinkled, almost equal in length to the ostium bursae. A long, heavily sclerotised cristate lateral ribbon present along the left side and extending into the basal part of the appendix bursae. Appendix bursae helicoid, quite long, with the distal end dilated slightly and the surface rough and rugose-wrinkled. Corpus bursae membranous, ovoid and four long signum-stripes present.

#### Diagnosis

The new species is the sister species of *A.changi*. They can be distinguished by the following characters: **a)** The size of the new species is somewhat larger, the forewing length of the type specimens of the new species ranges from 17 to 18 mm, for *A.changi*, it is 15 to 16 mm; **b)** The medial to distal section of the valva is broader than that of *A.changi*; **c)** The ampulla of the new species is distally narrower and more curved; **d)** In female genitalia, the distal end of the appendix bursae is smaller and narrower than that of *A.changi* and the surface is much rougher.

#### Etymology

The new species is dedicated to Professor Min Wang, who has carried out extensive work on Lepidoptera taxonomy in southern China.

#### Distribution

**Figure 5.**
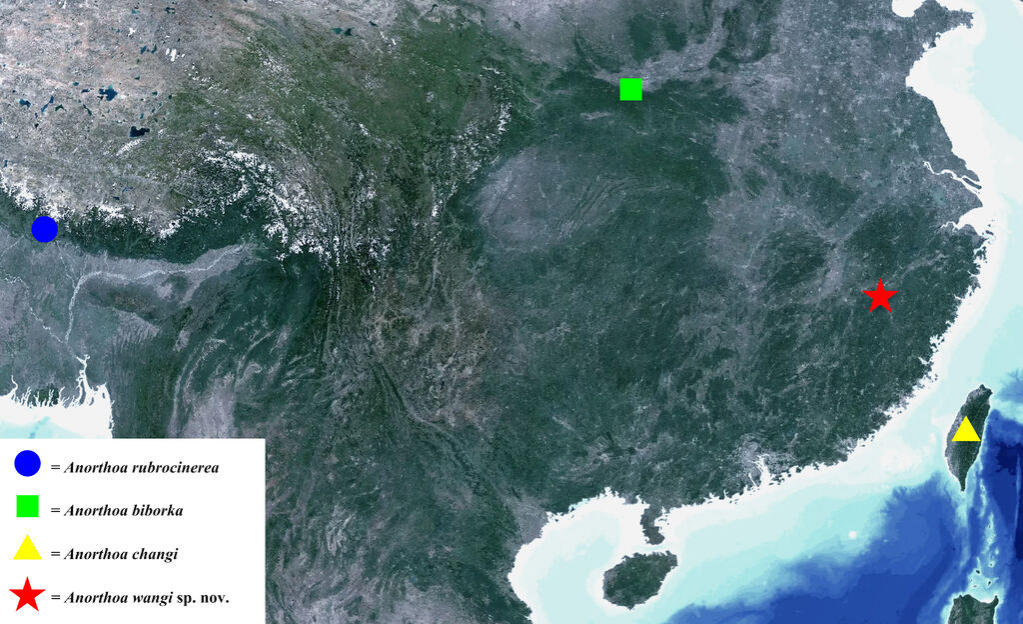
The type locality of the *rubrocinerea*-group.

Xianfengling, Wuyishan National Park, Wuyishan City, Fujian Province, south-eastern China (Fig. 5).

#### Biology

Seven adults were collected at the beginning of March in the Wuyi Mountain area.

## Supplementary Material

XML Treatment for Anorthoa wangi

## Figures and Tables

**Figure 1a.**
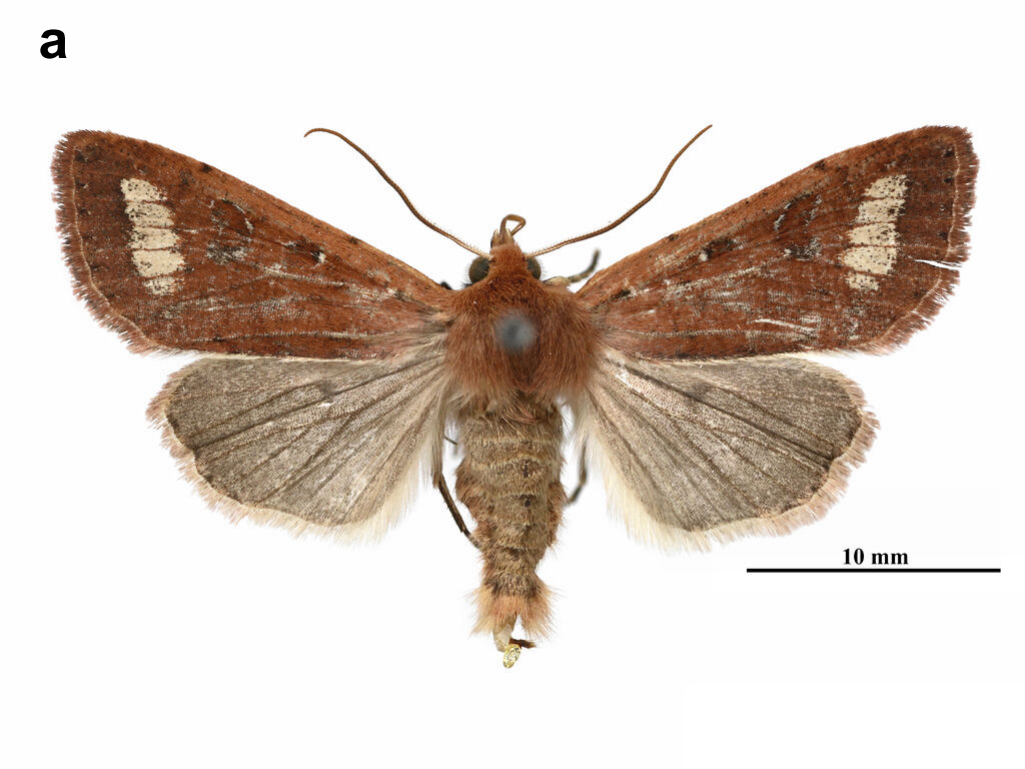
male, holotype, Fujian, China (coll. SCAU);

**Figure 1b.**
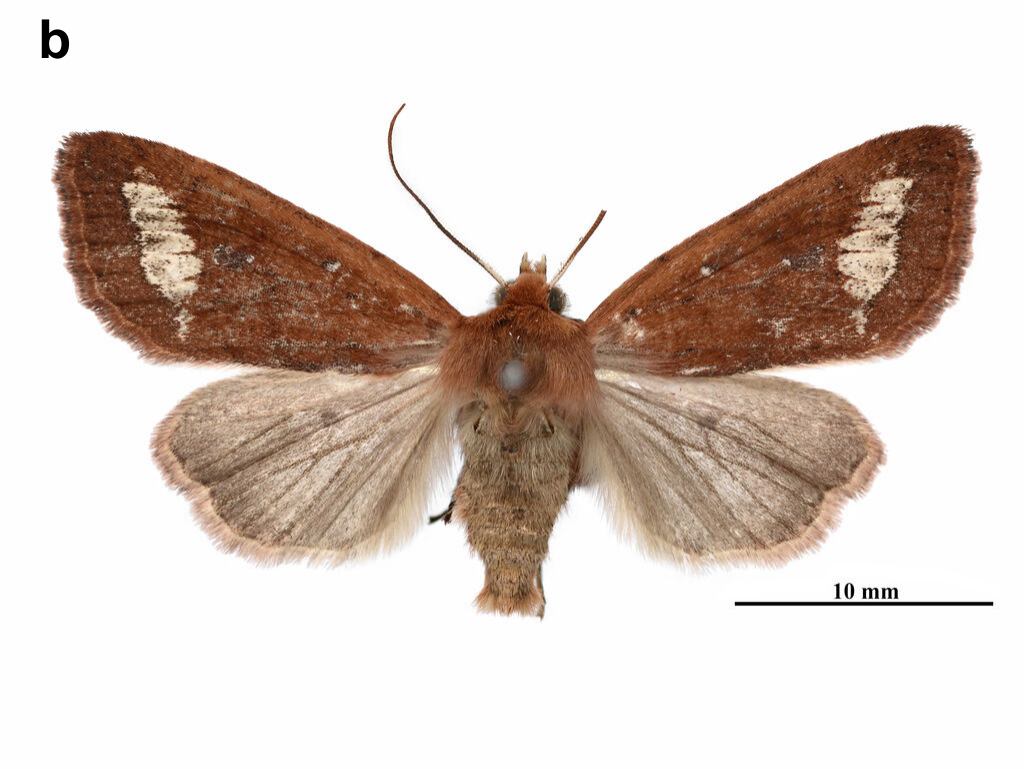
male, paratype, Fujian, China (coll. SCAU);

**Figure 1c.**
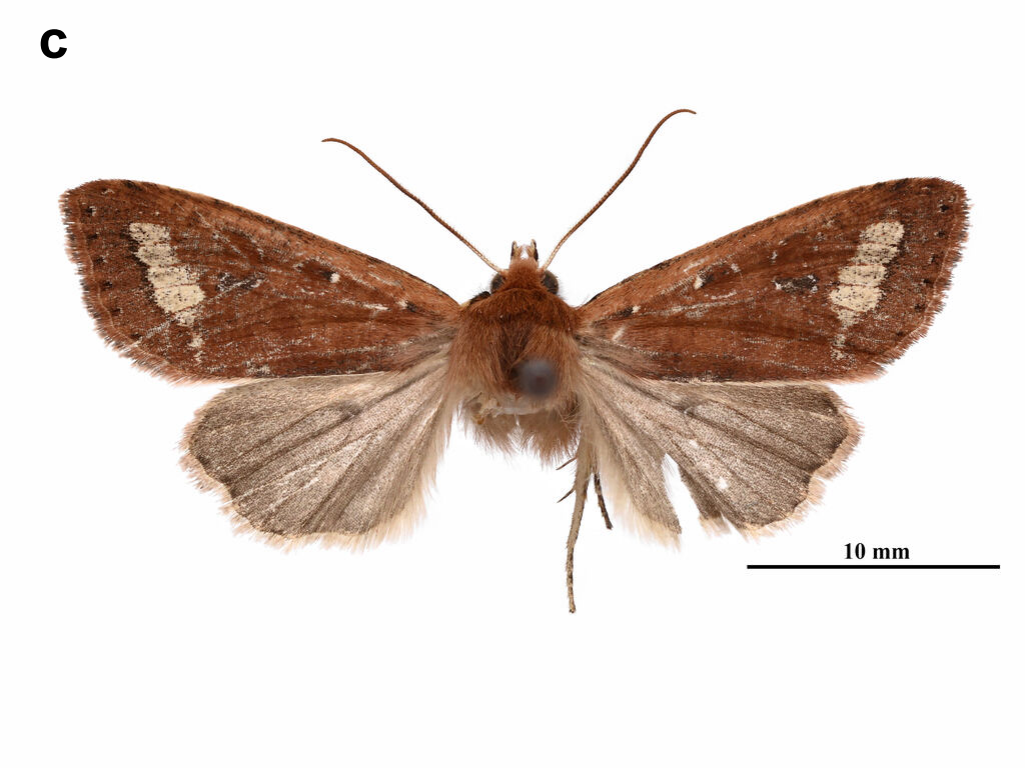
ditto;

**Figure 1d.**
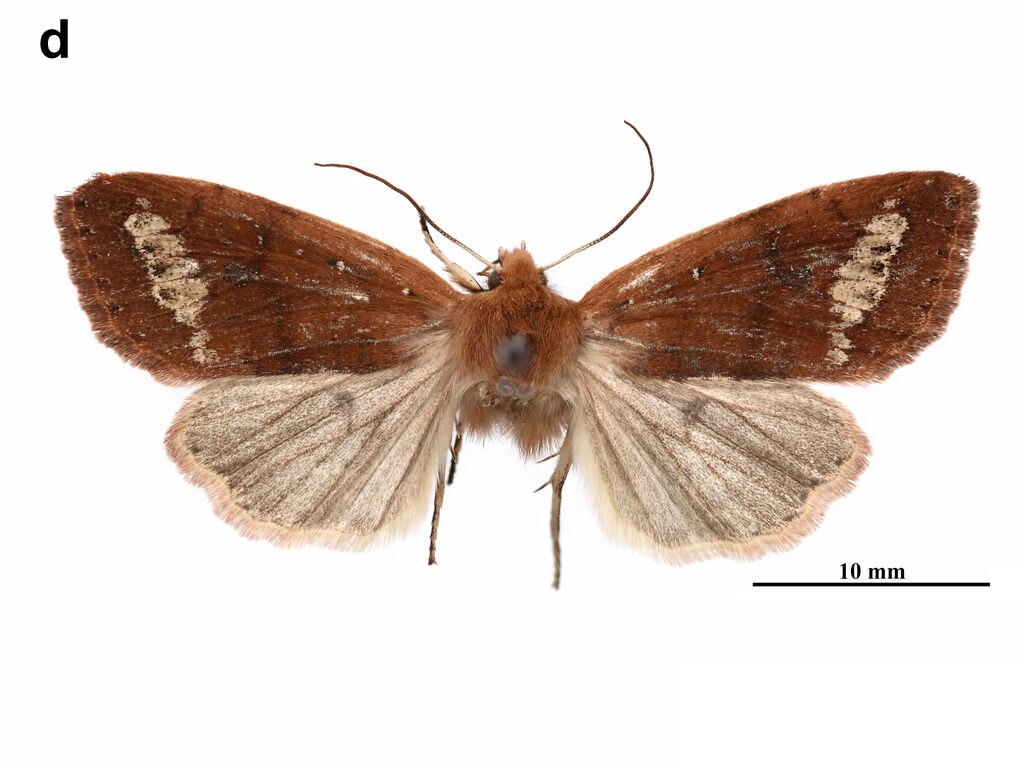
female, paratype, Fujian, China (coll. SCAU).

**Figure 3a.**
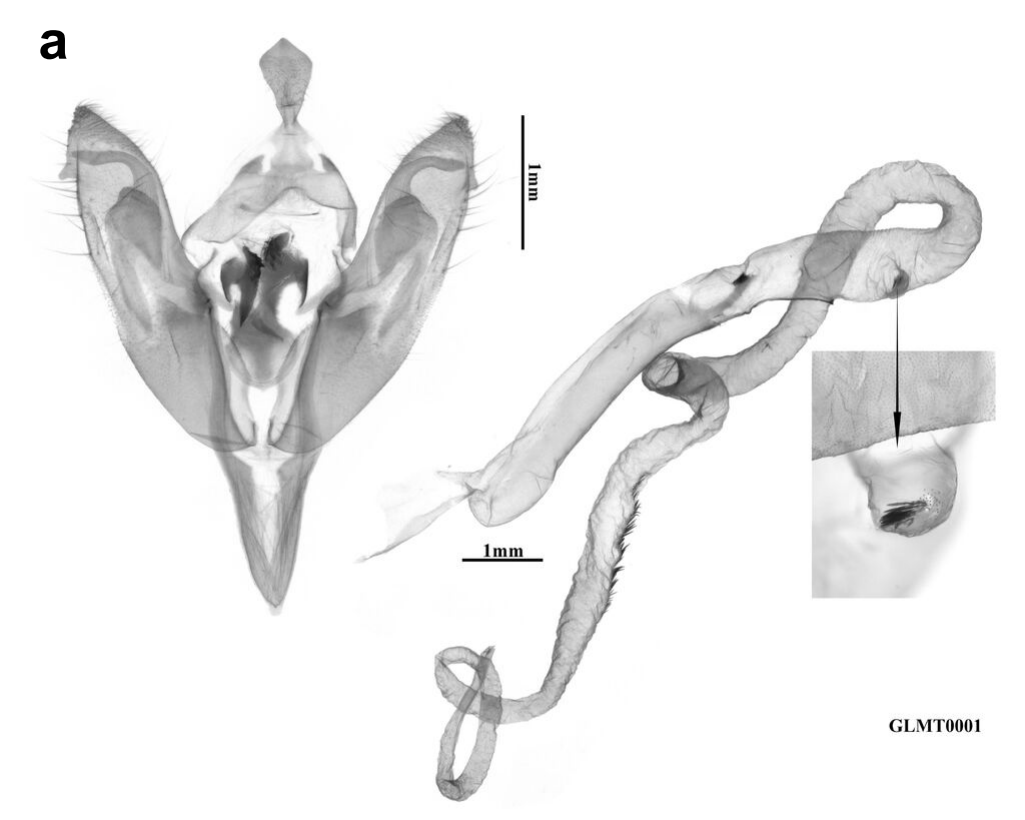
paratype, Fujian, China (prep. GLFJ0001);

**Figure 3b.**
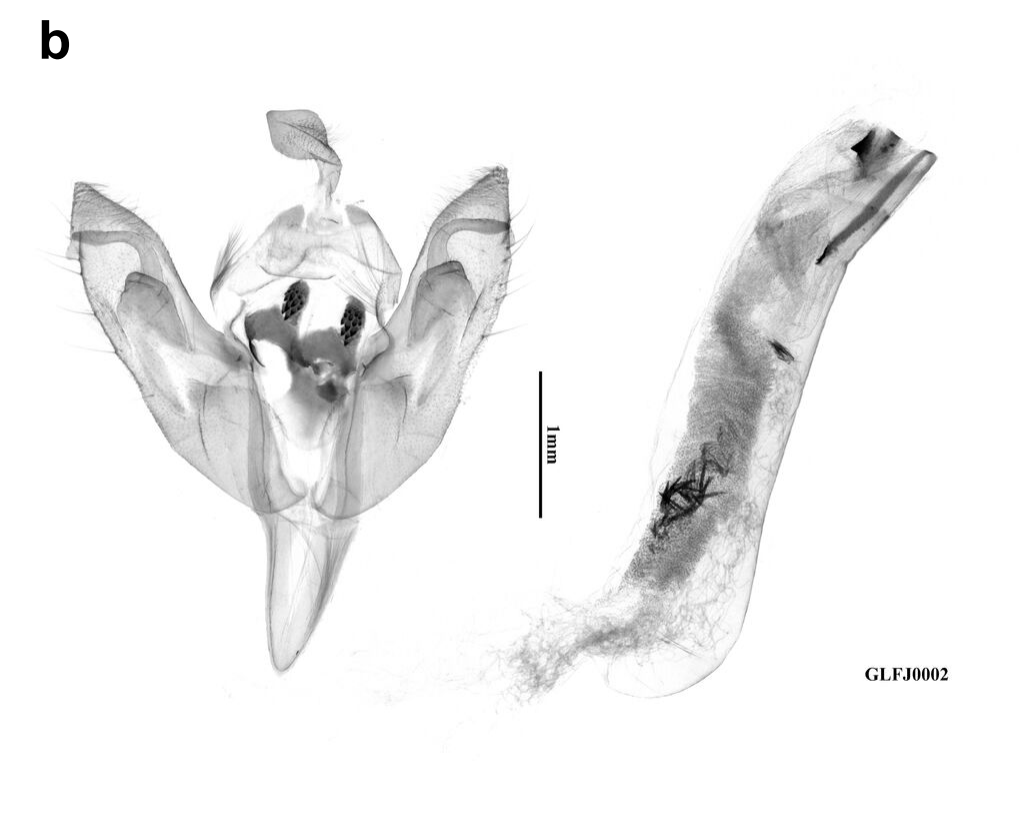
paratype, Fujian, China (prep. GLFJ0002).
